# Supplementary material for: Effectiveness of participatory community solutions strategy on improving household and provider health care behaviors and practices: A mixed-method evaluation
Source: PLoS One. 2020 Feb 5;15(2):e0228137. doi: 10.1371/journal.pone.0228137 (PMC7001957; doi:10.1371/journal.pone.0228137)
Supplement: S3 Appendix — This is an in-depth interview guide we used to interview the participants in our study. (DOCX) [file pone.0228137.s003.docx]

In-depth Interview Guide for PC-Solutions Strategy Qualitative Evaluation

# Introduction and Consent

Hello. My name is __________________. We are here on behalf of JSI/L10K to evaluate the effectiveness of the participatory community solutions (PC-Solution) strategy which has been implemented in eight PHCUs including your health center and cluster kebeles. This qualitative component of the study is primarily aimed to well understand the drivers and barriers to implementing the PC-Solutions strategy and major lessons learned.

Your facility was selected to be included in this study. We will be asking about the project implementation approaches, barriers and drivers of project implementation and lessons learned during implementation to deepen the understanding of the effectiveness of the project implementation in bringing impacts on MNH use.

By participating in this study, you can contribute with valuable information to improve the MNH care and practices. This in turn will increase the quality of life of mothers and babies. The information you share may also be provided to researchers for analyses, however, any reports that use your data will only present information in aggregate form so that neither you nor your facility can be identified. We will also inform you regarding the survey results.

We assure you that all information will be treated as confidential and will be kept in a secure environment. The name of your facility will not be identifiable and it will not be possible for people other than the researcher to link the information to your specific facility. We undertake to give you feedback of the results and outcomes, once the study has been completed.

If you want any additional information, or when you want to lodge any complaint or concern about any aspect of the research, you are welcome to contact the survey coordinator:

Mr. Gizachew Tadele Tiruneh

Addis Ababa, Ethiopia

Cell phone: +251 912003624

E-mail: gizt121@gmail.com

I agree that the research may be conducted in the facility under the terms and conditions indicated above.

Facility director’s signature: _________________ Date: ___________________

Interviewer's signature _________________ Date ______________________

# IDI questions to L10K staff

| Questionnaire ID: _____________ | |
| --- | --- |
| Date of Interview: [____\|____\| ______\|  dd \|mm \|yyyy | Interviewer Name: _______________ |
| Time interview Started: ____:____ | Time interview ended: ____:____ |

As you well remember, this participatory community quality improvement (QI) project has been implemented with the support from woreda health office and L10K since March 2016. Now we are going to ask you about the process of the implementation of the strategy.

1. Can you please explain what your responsibilities were during the implementation of the strategy?
2. How has the project been implemented? Was it innovative? Participatory? (explain)
3. Were you a part of the initial mapping exercise and/or consultative meeting? If yes, in your opinion, what was the benefit of the mapping exercise and/ or consultative meeting? What worked well? What could have been done differently?
4. What were the change ideas implemented in the PHCUs?
5. Have you participated in the review of the implementation of the change ideas with the health center staff/quality improvement team? How often? Was it regular? Have you participated in the woreda level review meetings? If yes, how could have been done differently?
6. What type of support did you provide to PHCUs? How often?
7. The analysis of the quantitative study showed significant intervention effects for improving utilization of MNH practices including early care seeking of women for ANC and provision of PNC by providers. However, the intervention effects were not statistically significant for first ANC, complete ANC, four and more ANC visits, ANC counseling score, satisfaction with delivery care score, skilled delivery, and disrespect and abuse during childbirth. Moreover, the BEmONC functions were not fully operational in most of the health centers.
8. What were the particular features (change ideas, activities, etc.) of the project that made a difference? Which ones were useful in improving quality of MNH care at facility and community level?
9. What do you think are the reasons for non-significant results?
10. In your opinion, what has facilitated or hindered the project to achieve its objectives and outcomes? What worked best for whom, why and when?
11. Were there any consequences of the strategy? Both adverse consequences and positive behavioral changes?
12. What were the major challenges you were facing in relation to conducting this QI project? (Regularity of the QI events, capacity, workload, community participation, etc.)
13. How could the strategy be better implemented so that it could be more effective?
14. What were the biggest lessons in this endeavor?

**Thank you very much for your time, indeed!!!**

# IDI questions to health center staff

| Questionnaire ID: _____________ | |
| --- | --- |
| Date of Interview: [____\|____\| ______\|  dd \|mm \|yyyy | Interviewer Name: _______________ |
| Time interview Started: ____:____ | Time interview ended: ____:____ |

As you well know, this participatory community quality improvement (QI) project has been implemented with the support from woreda health office and JSI/L10K since March 2016. Now we are going to ask you about the process of the implementation of the strategy in your PHCU.

1. What is your current position? How long have you been in this position?
2. Can you explain what your responsibilities are here at the health center?
3. What were your specific responsibilities in relation to implementation of the strategy?
4. How has the project been implemented? Was it innovative? Participatory?
5. Did you participate in the initial mapping exercise that L10K conducted with the PHCU, HEWs and the community? If yes, in your opinion, what was the benefit of the mapping exercise? What worked well? What could have been done differently?
6. Did you participate in the consultative meeting that L10K conducted with PHCU and the community? If yes, in your opinion, what was the benefit of the consultative meeting? What worked well? What could have been done differently?
7. Following the consultative meeting, what were the change ideas that were implemented in this health center?
8. Did you review the process of implementation of these change ideas with the health center staff/quality improvement team? How often?
9. Have you observed any changes as the result of the implementation of the project? What were the particular features (change ideas, activities, etc.) of the project that made a difference? Which ones were useful in improving quality of MNH care at facility and community level?

**Probe:** The analysis of the quantitative study showed significant intervention effects for improving utilization of MNH practices including early care seeking of women for ANC and provision of PNC by providers. However, the intervention effects were not statistically significant for first ANC, complete ANC, four and more ANC visits, ANC counseling score, satisfaction with delivery care score, skilled delivery, and disrespect and abuse during childbirth. Moreover, the BEmONC functions were not fully operational in most of the health centers.

1. What do you think are the reasons for success and for non-significant results?
2. Have you participated in the woreda level review meetings that the PHCU conducted with HEWs, Woreda staff, L10K staff, and the community? If yes, how could have been done differently?
3. Have you facilitated in the kebele level QI cycles that the HEWs and community volunteers participated? How often? What were the change ideas implemented at the kebele level?
4. What support from the L10K team did your PHCU receive? How often did an L10K team member come to visit you? What type of support did L10K provide you?
5. In your opinion, what has facilitated or hindered the project to achieve its objectives and outcomes? What worked best for whom, why and when?
6. Were there any consequences of the strategy? Both adverse consequences and positive behavioral changes?
7. What were the major challenges you were facing in relation to conducting this QI project? (Regularity of the QI events, capacity, workload, community participation, etc.)
8. How could the strategy be better implemented so that it could be more effective?
9. What were the biggest lessons in this endeavor?
10. Is there anything else you would like to tell us?

**Thank you very much for your time, indeed!!!**

# IDI questions to HEWs

| Questionnaire ID: _____________ | |
| --- | --- |
| Date of Interview: [____\|____\| ______\|  dd \|mm \|yyyy | Interviewer Name: _______________ |
| Time interview Started: ____:____ | Time interview ended: ____:____ |

As you well know, this participatory community quality improvement (QI) project has been implemented with the support from woreda health office and JSI/L10K since March 2016. Now we are going to ask you about the process of the implementation of the strategy in your Kebele.

1. How long have you served as an HEW?
2. What were your specific responsibilities in relation to implementation of the strategy?
3. How has the project been implemented? Was it innovative? Participatory?
4. Did you participate in the initial mapping exercise that L10K conducted with the PHCU and the community? If yes, in your opinion, what was the benefit of the mapping exercise? What worked well? What could have been done differently?
5. Did you participate in the consultative meeting that L10K conducted with PHCU and the community? If yes, in your opinion, what was the benefit of the consultative meeting? What worked well? What could have been done differently?
6. Following the consultative meeting, what were the change ideas implemented in your kebele?
7. Did you review the process of implementation of these change ideas with the HDAs/kebele cabinets? How often?
8. Have you observed any changes as the result of the implementation of the project? What were the particular features (change ideas, activities, etc.) of the project that made a difference? Which ones were useful in improving quality of MNH care at facility and community level?
9. Have you participated in the woreda level review meetings that the PHCU conducted with Woreda staff, L10K staff, and the community? If yes, how could have been done differently?
10. What support from the L10K and health center staff did you receive? How often did they come to visit you? What type of support did they provide you?
11. In your opinion, what has facilitated or hindered the project to achieve its objectives? What worked best for whom, why and when?
12. Were there any consequences of the strategy? Both adverse consequences and positive behavioral changes?
13. What were the major challenges you were facing in relation to conducting this QI project? (Regularity of the QI events, capacity, workload, community participation, etc.)
14. How could the strategy be better implemented so that it could be more effective?
15. What were the biggest lessons in this endeavor?
16. Is there anything else you would like to tell us?

**Thank you very much for your time, indeed!!!**

# IDI questions to HDAs

| Questionnaire ID: _____________ | |
| --- | --- |
| Date of Interview: [____\|____\| ______\|  dd \|mm \|yyyy | Interviewer Name: _______________ |
| Time interview Started: ____:____ | Time interview ended: ____:____ |

As you well know, this participatory community quality improvement (QI) project has been implemented with the support from woreda health office and JSI/L10K since March 2016. Now we are going to ask you about the process of the implementation of the strategy in your community.

1. How long have you served as HDA?
2. In relation to implementation of the strategy, what were your specific responsibilities?
3. Thinking of the women in your community, what are the main barriers for women to get early ANC, deliver in a facility, and receive PNC? What are the major challenges for you to let women in your community to get early ANC, delivery in the facility and receive early PNC?
4. Were you a part of the initial mapping exercise that L10K conducted with the PHCU and the community? If yes, in your opinion, what was the benefit of the mapping exercise? What worked well? What could have been done differently? Did you feel you had a voice in the process?
5. Were you a part of the consultative meeting that L10K conducted with PHCU and the community? If yes, in your opinion, what was the benefit of the consultative meeting? What worked well? What could have been done differently? Did you feel you had a voice at the meeting?
6. Following the consultative meeting, what were the change ideas implemented in your kebele?
7. Have you participated in the review the process of implementation of these change ideas with the HEWs? How often? Was it regular? What support did the HEW provide to you? What additional support could she provide to you?
8. Have you seen changes regarding women’s use early ANC, facility delivery, and early PNC care in your community? What were the particular features (change ideas, activities, etc.) of the project that made a difference? Which ones were useful in improving quality of MNH care at facility and community level?
9. Have you participated in the woreda level review meetings that the PHCU conducted with Woreda staff, L10K staff, and the community? If yes, what happened during these meetings? What types of issues were discussed?
10. In your opinion, what has facilitated or hindered the project to achieve its objectives (i.e. to let women seek care and practice behaviour)?
11. What were the major challenges you were facing in relation to doing this QI project? (Regularity of the QI events, capacity, workload, community participation, etc.)
12. Is there anything else you would like to tell us?

**Thank you very much for your time, indeed!!!**
