# Supplementary material for: Effectiveness of participatory community solutions strategy on improving household and provider health care behaviors and practices: A mixed-method evaluation
Source: PLoS One. 2020 Feb 5;15(2):e0228137. doi: 10.1371/journal.pone.0228137 (PMC7001957; doi:10.1371/journal.pone.0228137)
Supplement: Attachment — Submitted filename: Response to Reviewers.docx [file pone.0228137.s005.docx]

**Point-by-point response to reviewer/editorial**

**Version 1**

J**ournal:** PLOS ONE

**Title:** " Effectiveness of Participatory Community Solutions Strategy on Improving Household and Provider Health Care Behaviors and Practices: A Mixed Method Evaluation (PONE-D-19-15140)”

*The authors would like to appreciate and thank the reviewers for the constructive comments.*

*Our point-by-point responses to the reviewers are below each of the comments in italics. We also make sure that this version of the manuscript conforms the journal style.*

Journal Requirements:

When submitting your revision, we need you to address these additional requirements.

1. Please ensure that your manuscript meets PLOS ONE's style requirements, including those for file naming. The PLOS ONE style templates can be found at

<http://www.journals.plos.org/plosone/s/file?id=wjVg/PLOSOne_formatting_sample_main_body.pdf> and <http://www.journals.plos.org/plosone/s/file?id=ba62/PLOSOne_formatting_sample_title_authors_affiliations.pdf>

*Comment well taken.*

2. Data availability. Please note that authors should not be the sole named individuals responsible for ensuring data access. If these data cannot be publicly deposited or included in the supporting information, e.g. due to patient privacy, legal reasons, or being provided by a third party, please explain why and explain how researchers may access them via a named data access committee or named ethics committee.

*Comment well taken. And in this version, the data used for this analysis are submitted as supplementary material, S1 Appendix.*

3. Please specify in your financial disclosure whether the funders played any role in the study.  This information should be included in your cover letter; we will change the online submission form on your behalf.

*Thanks for reminding us. The following paragraph included in the financial disclosure section. “The article write-up and publication fee was supported by the Bill & Melinda Gates Foundation, Grant Number OPP1131042. JSI Research & Training Institute, Inc. has provided us support in the form of salaries for authors [GT, AK, NZ, WB]. However, any of the funders did not have role in study design, data collection and analysis, decision to publish, or preparation of the manuscript.”*

4. Please specify whether an interview guide was used to interview the participants in your study. If yes, please describe and/or include a copy as a Supporting Information file.

*Comment well taken. Interview guides were used to capture the data and in this version, interview guides are included as supplementary files,* *S3 Appendix.*

5. Please include additional information regarding the survey used in the study and ensure that you have provided sufficient details that others could replicate the analyses. For instance, if you developed a questionnaire as part of this study and it is not under a copyright more restrictive than CC-BY, please include a copy, in both the original language and English, as Supporting Information.

*Comment well taken. Standard questionnaire was used to capture the data and in this version, the questionnaire is included as supplementary files, S2 Appendix.*

6. Please carefully proof read your manuscript. For example, there is a missing space in the abstract “…November 2017.Propensity scores…”.

*Well noted.*

7. Please amend your current ethics statement to address the following concerns:   
a) Did participants provide their written or verbal informed consent to participate in this study?  
b) If consent was verbal, please explain:  
i) Why was written consent not obtained?   
ii) How did you record/document participant consent?  
iii) Did the ethics committees/IRBs approve this consent procedure?

*The ethics statement now addressed the issues raised and it reads as follows; “Ethical clearances for the surveys were obtained from the ethical review boards of Amhara, Oromia, SNNP, Tigray Regional Health Bureaus, and JSI. All the study participants were informed about the purpose of the study; benefits and hazards of the study were explained to all study participants, and each participant was notified of their right to opt out when responding to questions. Verbal Consent was sought and documented before conducting any interviews. If the respondent was less than 18 years old, then consent was sought from her husband or guardian. Majority of the respondents were not expected to be able to read or write; as such, written consent was not sought. If the respondent agreed to be interviewed after listening to the consent statement, the interviewer marked the questionnaire as consent given below the consent statement and then signed below that. The interviewer continued with the interview only after receiving and documenting the consent. The survey protocol submitted to the ethical review committee included the study questionnaire with the consent statement that described the consent obtaining procedure and it was approved by the committee. Moreover, the information obtained from the research participants were kept in private (codes were used during reporting of the IDI quotes).”*

8. Thank you for stating the following in the Competing Interests section:

"The authors have declared that no competing interests exist."

We note that one or more of the authors are employed by a commercial company:  JSI Research & Training Institute, Inc

a. Please provide an amended Funding Statement declaring this commercial affiliation, as well as a statement regarding the Role of Funders in your study. If the funding organization did not play a role in the study design, data collection and analysis, decision to publish, or preparation of the manuscript and only provided financial support in the form of authors' salaries and/or research materials, please review your statements relating to the author contributions, and ensure you have specifically and accurately indicated the role(s) that these authors had in your study. You can update author roles in the Author Contributions section of the online submission form.

Please also include the following statement within your amended Funding Statement.

“The funder provided support in the form of salaries for authors [insert relevant initials], but did not have any additional role in the study design, data collection and analysis, decision to publish, or preparation of the manuscript. The specific roles of these authors are articulated in the ‘author contributions’ section.”

If your commercial affiliation did play a role in your study, please state and explain this role within your updated Funding Statement.

b. Please also provide an updated Competing Interests Statement declaring this commercial affiliation along with any other relevant declarations relating to employment, consultancy, patents, products in development, or marketed products, etc.

Within your Competing Interests Statement, please confirm that this commercial affiliation does not alter your adherence to all PLOS ONE policies on sharing data and materials by including the following statement: "This does not alter our adherence to  PLOS ONE policies on sharing data and materials.” (as detailed online in our guide for authors <http://journals.plos.org/plosone/s/competing-interests>) . If this adherence statement is not accurate and  there are restrictions on sharing of data and/or materials, please state these. Please note that we cannot proceed with consideration of your article until this information has been declared.

c. Please include both an updated Funding Statement and Competing Interests Statement in your cover letter. We will change the online submission form on your behalf.

Please know it is PLOS ONE policy for corresponding authors to declare, on behalf of all authors, all potential competing interests for the purposes of transparency. PLOS defines a competing interest as anything that interferes with, or could reasonably be perceived as interfering with, the full and objective presentation, peer review, editorial decision-making, or publication of research or non-research articles submitted to one of the journals. Competing interests can be financial or non-financial, professional, or personal. Competing interests can arise in relationship to an organization or another person. Please follow this link to our website for more details on competing interests: <http://journals.plos.org/plosone/s/competing-interests>

*Comment well taken. And the Funding Statement and Competing Interests Statement is updated as follows: “The article write-up and publication fee was supported by the Bill & Melinda Gates Foundation, Grant Number OPP1131042. The authors have been working for JSI Research & Training Institute, Inc., a commercial company. We declared that this commercial affiliation does not alter our adherence to PLOS ONE policies on sharing data and materials. JSI Research & Training Institute, Inc. has provided us support in the form of salaries for authors [GT, AK, NZ, WB]. However, any of the funders did not have role in study design, data collection and analysis, decision to publish, or preparation of the manuscript.”*

9.  Thank you for including your ethics statement:
"Ethical clearances for the surveys were obtained from the ethical review boards of Amhara, Oromia, SNNP, Tigray Regional Health Bureaus, and JSI. All the study participants were informed about the purpose of the study; benefits and hazards of the study were explained to all study participants, and each participant was notified of their right to opt out when responding to questions. Consent was sought before conducting any interviews. Moreover, the information obtained from the research participants were kept in private (codes were used during reporting of the IDI quotes)."

a. Please amend your current ethics statement to confirm that your named institutional review board or ethics committee specifically approved this study.

b. Once you have amended this/these statement(s) in the Methods section of the manuscript, please add the same text to the “Ethics Statement” field of the submission form (via “Edit Submission”).

For additional information about PLOS ONE ethical requirements for human subjects research, please refer to <http://journals.plos.org/plosone/s/submission-guidelines#loc-human-subjects-research>.

*The ethics statement now amended as follows; “Ethical clearances for the surveys were obtained from the ethical review boards of Amhara, Oromia, SNNP, Tigray Regional Health Bureaus, and JSI. All the study participants were informed about the purpose of the study; benefits and hazards of the study were explained to all study participants, and each participant was notified of their right to opt out when responding to questions. Verbal Consent was sought and documented before conducting any interviews. If the respondent was less than 18 years old, then consent was sought from her husband or guardian. Majority of the respondents were not expected to be able to read or write; as such, written consent was not sought. If the respondent agreed to be interviewed after listening to the consent statement, the interviewer marked the questionnaire as consent given below the consent statement and then signed below that. The interviewer continued with the interview only after receiving and documenting the consent. The survey protocol submitted to the ethical review committee included the study questionnaire with the consent statement that described the consent obtaining procedure and it was approved by the committee. Moreover, the information obtained from the research participants were kept in private (codes were used during reporting of the IDI quotes).”*

10. We note that you have included the phrase “data not shown” in your manuscript. Unfortunately, this does not meet our data sharing requirements. PLOS does not permit references to inaccessible data. We require that authors provide all relevant data within the paper, Supporting Information files, or in an acceptable, public repository. Please add a citation to support this phrase or upload the data that corresponds with these findings to a stable repository (such as Figshare or Dryad) and provide and URLs, DOIs, or accession numbers that may be used to access these data. Or, if the data are not a core part of the research being presented in your study, we ask that you remove the phrase that refers to these data.

*Comment well taken. And Table is inserted as source in the text.*

11. We note that you have indicated that data from this study are available upon request. PLOS only allows data to be available upon request if there are legal or ethical restrictions on sharing data publicly. For more information on unacceptable data access restrictions, please see <http://journals.plos.org/plosone/s/data-availability#loc-unacceptable-data-access-restrictions>.

In your revised cover letter, please address the following prompts:

a) If there are ethical or legal restrictions on sharing a de-identified data set, please explain them in detail (e.g., data contain potentially sensitive information, data are owned by a third-party organization, etc.) and who has imposed them (e.g., an ethics committee). Please also provide contact information for a data access committee, ethics committee, or other institutional body to which data requests may be sent.

b) If there are no restrictions, please upload the minimal anonymized data set necessary to replicate your study findings as either Supporting Information files or to a stable, public repository and provide us with the relevant URLs, DOIs, or accession numbers. For a list of acceptable repositories, please see <http://journals.plos.org/plosone/s/data-availability#loc-recommended-repositories>.

We will update your Data Availability statement on your behalf to reflect the information you provide.

*The survey dataset we used for the analysis is provided as supplementary information (S1 Appendix).*

Additional Editor Comments (if provided):

I commend the authors for this important work and hope they can sufficiently address the comments from myself and Reviewer #1.

Major Comments:
The qualitative results are difficult to interpret given the lack of detail about the methods of data collection and analysis. Please give more detail about the qualitative portion of the study, referring to PLOS ONE guidelines below:
Qualitative research studies should be reported in accordance to the [Consolidated criteria for reporting qualitative research (COREQ) checklist.](http://intqhc.oxfordjournals.org/content/19/6/349.long) Further reporting guidelines can be found in the [Equator Network's Guidelines for reporting qualitative research](http://www.equator-network.org/?post_type=eq_guidelines&eq_guidelines_study_design=qualitative-research&eq_guidelines_clinical_specialty=0&eq_guidelines_report_section=0&s=)
The qualitative section “improved service utilization and qualitative of care” does not seem to add much to the overall study. What value does perceived effectiveness add if there is quantitative data to more objectively assess effectiveness? How do we know the answers of the WDA to their funders were not influenced by social desirability bias?

*Comment well taken. The qualitative sections are moved as suggested by Reviewer #1 and the COREQ checklist is followed. Moreover, the possibility of recall and social-desirability bias are presented as limitations of this paper in the Discussion section.*

I agree with Reviewer #1’s comment about the multiple outcomes. Was there a primary outcome that was chosen prior to the trail as the indicator of intervention success, or one that the intervention was most focused on achieving? Or, among the many indicators, was there any statistical adjustment for multiple outcomes?

*The project was designed to influence multiple RMNCH indicators including antepartum, intrapartum, and postpartum. As, it was clearly indicated in the project document as well as the research protocol, below are the list of indicators the project intended to influence;*

***Antenatal care:*** *antenatal care (ANC); complete ANC (BP measured and urine & blood tested); ANC in 1st trimester; 4 or more ANC visits (ANC 4+), ANC in 1st & last trimester*

***Perinatal care:*** *Institutional delivery/skilled health personnel; institutional delivery among the lowest wealth quintile; MgSO4 given for pre-eclampsia; Oxytocin given during 3rd stage of labor; partograph use rate; met need for emergency obstetric & newborn care (EmONC); signal functions of basic EmONC (BEmONC); still birth rates.*

***Postnatal & newborn care:*** *newborn care, neonatal infection; neonatal asphyxia managed; postnatal care (PNC) in 48 hours; complete PNC in 48 hours.*

*As such, there was no adjustment made* for multiple hypothesis testing.

The literature review could be more robust, especially when referencing evidenced-based intervention (e.g. kangaroo care), please take care to add appropriate citations.

*Comment well acknowledged. Availability of evidence-based, low-cost, life-saving MNH interventions as well as their impact and implementation challenges are now presented in the Introduction section, paragraph 1 and 2 of this version.*

Minor Comments:
Abstract
Please edit for grammar: line 21 “We evaluated the effects of this strategy using a mixed methods research” and again in line 80 (take out “a” or rephrase to: “a mixed-methods research strategy)
Line 25 A general reader would not know what is meant by “kebeles”. Please define or reword

*Thanks for the comments. Now, it is corrected*

Intro
Grammar: line 45 “there is high fall out rates”

*Thanks for the comments. Now, it is corrected*
Table 2
Readiness to perform signal functions
- Please indicate if the “availability of…” indicators use a dichotomous (yes/no) response, or if there is some other measure of levels of availability

*Comments well acknowledged and indicated as yes/no response.*

Lines 238-248: Please justify your choice of these variables as potential confounders, and if appropriate add references
Please justify with a reference or other explanation the choice of a 0.2 p-value for the logit stepwise backward selection

*Potential confounders are identified from previous studies and these variables are indicated in the Introduction section of the manuscript, Page 4, line 54-59.*

*Thanks a lot for the comments. Scholars recommended to match variables on the logit of the propensity score using calipers of width equal to 0.2 of the standard deviation of the logit of the propensity score when estimating differences in means or risk differences. Relevant references are now cited (Austin, 2009, 2011)., page 19, line 300.*

Line numbers stop on page 31.

*Thanks for the comments. Now we make the line number continuous.*

There appears to be a missing quotation in the middle of page 34

*Sure, it was missed. Now, inserted.*

Middle of page 36: Please give some more detail about the “previous study in Ethiopia” and how the results of the present study align with it.

*Well acknowledged and addressed as suggested.*

Middle of page 38: “synthesized to unpack” sounds contradictory

*Noted and corrected*

Throughout:
Recommend italicizing “woreda” and other local terms
Be consistent in capitalization for Family Conversation and Birth Notification

*Comments well taken*

**Comments to the Author**

1. Is the manuscript technically sound, and do the data support the conclusions?

Reviewer #1: Partly

*Major revisions made on the data presentation, particularly the qualitative section.*

2. Has the statistical analysis been performed appropriately and rigorously?

Reviewer #1: Yes

  3. Have the authors made all data underlying the findings in their manuscript fully available?

Reviewer #1: No

*Now included as supplementary file, S1 Appendix.*

4. Is the manuscript presented in an intelligible fashion and written in standard English?

Reviewer #1: No

*We improved the language in this version.*

5. Review Comments to the Author

Reviewer #1: I commend the authors on synthesising results of this important evaluation of a complex intervention strategy in multiple regions of Ethiopia. I wish to offer the following substantive and minor comments.

Substantive comments:

1. This is an evaluation of a complex intervention with multiple components. It would be helpful to include a table with all intervention components, their timeframes and geographical coverage in the methods section.

*Comment well acknowledged. Table 1 is inserted in this version.*

2. Strengthen and move qualitative sections:

In this article, the qualitative sections are used to: (a) help describe the interventions; (b) highlight barriers and facilitators to implementation; and (c) derive lessons for future implementation. Unfortunately all this is done without presenting any actual qualitative data. I would recommend simplifying this and using a table or diagramme to present the content of interventions (the innovation and implementation mechanisms, line 287 onwards) and removing the sections where qualitative data on intervention implementation are summarised. I would also recommend condensing sections on lessons learned or providing data to support the statements made there. The most valuable use of the qualitative data is in the section on barriers and facilitators. This can be strengthened through the use of quotes and reporting of discrepant cases, otherwise they is no actual qualitative analysis, just a brief summary of views from programme insiders.

Consider moving remaining qualitative sections (barriers and facilitators) after the DID results so they help explain your results. If you do not feel like you can do the above, I would recommend publishing the qualitative analysis in a separate article where you can really do it justice.

Finally, I would remove the reference to themes 'emerging' from the data as it seems that data were mostly coded in response to the research questions.

*Comment well taken. Detailed description of the intervention is now presented in Table 1. Moreover, the qualitative sections are moved as suggested. More quotes are added to strengthened the evidence of the findings.*

3. Background - You could state what the quantitative research questions were/was, for balance. At the moment you also list the qualitative questions.

*Comment well taken. We convinced that objectives can clearly convey the intended message. As such, in this version, only objectives of both the qualitative and quantitative studies are presented and for balance, we dropped the research questions of the qualitative study.*

4. Was the impact evaluation registered? If so where is this registration number?

*This evaluation was indicated in the project document. Following the project design, a research protocol was developed. The evaluation protocol was not registered in any of the online registries. The authors can submit a draft protocol as supplementary material, if needed.*

5. The quantitative impact evaluation has multiple outcomes, was there an a priori data analysis plan, and was any adjustment made for multiple hypothesis testing?

*The project was designed to influence the following antepartum, intrapartum, and postpartum indicators:*

***Antenatal care:*** *antenatal care (ANC); complete ANC (BP measured and urine & blood tested); ANC in 1st trimester; 4 or more ANC visits (ANC 4+), ANC in 1st & last trimester, tested for Syphilis.*

***Perinatal care:*** *Institutional delivery; skilled birth attendance; MgSO4 given for pre-eclampsia; prophylactic uterotonics; antibiotics for pPRom; antenatal corticosteroids; partograph use rate; met need for emergency obstetric & newborn care (EmONC); signal functions of basic EmONC (BEmONC); still birth rates.*

***Postnatal & newborn care:*** *Immediate drying the baby; immediate breastfeeding; kangaroo mother care of facility deliveries; safe cord care; thermal care; neonatal resuscitation; neonatal infection management; maternal postnatal care (PNC); complete maternal PNC; neonatal postnatal care (PNC); complete neonatal PNC; FP counseling provided during PNC.*

*This evaluation was based on the project document. As such, there was no any adjustment made to test multiple hypothesis after or before data collection.*

7. Abstract and Results - Your data do not support that "receiving early antenatal care between baseline and follow-up surveys in the intervention area is attributable to the strategy" because the confidence interval includes 0, i.e. the possibility of no effect. This needs to be removed.

*Comment well taken.*

8. Discussion - the main reason for lack of effect on most MNCH indicators aside from skilled care at birth and receipt of PNC for mother and baby was the minimal intervention duration time. Does anything else explain the results?

*Comment well take. And in this version, we supplemented the possible reasons from the qualitative study for low intervention coverage. Page 43, line 706-9.*

9. Discussion - A key limitation of the qualitative work is the lack of sampling community members not linked to the project, i.e. non-project-based staff, HEWs or HDAs. Is there a reason why you did not speak with mothers about their views of what changed and what did not?

*The main objective of the qualitative study was to describe the process of implementation and understand the complex participatory QI process; highlight barriers and facilitators of the implementation and scalability of the intervention. We believe active community level intervention players, community volunteers (WDAs) and front line health workers (HEWs) would be the key informants for the purpose. Accordingly, we did not interview mothers, direct beneficiaries for the project.*

10. For your consideration - I would avoid the use of the term 'innovations' - community interventions like family conversations and facility or health systems-levels interventions like QI or PDSA cycles are not really 'innovations'. They may be in this context, but there is a long history of their use in multiple contexts. The donor-favoured term 'innovation' is a little tiring; can we just call an intervention an intervention?

*Comment well taken.*

11. Conflict of interest: the senior author of this article is from the Gates Foundation, which also funded the writing of this article and, critically, the interventions being evaluated. The role of Foundation-paid staff in writing up/editing results from their own projects needs to be explained or declared more clearly as a conflict of interest. Please see recent demands for transparency in such situations: <https://gh.bmj.com/content/4/3/e001746>

The senior author of this manuscript has been working for JSI Research and Training Institute Inc. during the design, field work, and report-up of this manuscript. He recently left JSI and joined Gates Foundation, In February 2019.

*We updated the Funding Statement and Competing Interests Statement as follows: “The article write-up and publication fee was supported by the Bill & Melinda Gates Foundation, Grant Number OPP1131042. The authors have been working for JSI Research & Training Institute, Inc., a commercial company. We declared that this commercial affiliation does not alter our adherence to PLOS ONE policies on sharing data and materials. JSI Research & Training Institute, Inc. has provided us support in the form of salaries for authors [GT, AK, NZ, WB]. However, any of the funders did not have role in study design, data collection and analysis, decision to publish, or preparation of the manuscript.”*
