# Supplementary material for: Effectiveness of participatory community solutions strategy on improving household and provider health care behaviors and practices: A mixed-method evaluation
Source: PLoS One. 2020 Feb 5;15(2):e0228137. doi: 10.1371/journal.pone.0228137 (PMC7001957; doi:10.1371/journal.pone.0228137)
Supplement: Attachment — Submitted filename: Response to Reviewers.docx [file pone.0228137.s006.docx]

**Point-by-point response to reviewer/editorial**

**Version 2**

J**ournal:** PLOS ONE

**Title:** " Effectiveness of Participatory Community Solutions Strategy on Improving Household and Provider Health Care Behaviors and Practices: A Mixed Method Evaluation (PONE-D-19-15140R1)”

*The authors would like to appreciate and thank the editor for the constructive comments.*

*Our point-by-point responses are below each of the comments in italics. We also make sure that this version of the manuscript conforms the journal style. The manuscript is reviewed by a native speaker, Julie Ray.*

Intro

Third line- “being” can be deleted, can be simply “care provided”

First line of second paragraph: specify what “it” is

*Thank you so much for the comments. The language is now edited with a native speaker and grammatically improved.*

Last paragraph of the intro could be improved. In the line proceeding, you indicate that the study was mixed-methods, and then only describe the qualitative component in the next paragraph. Be clear as if you are presenting results from the quantitative sections or not. If so, state the overall objective of the mixed-methods study as well as the quantitative and qualitive sections.

*Thanks a lot for the valuable comments. The quantitative component is now described (line 94-96, page 6).*

Methods

More detail on the qualitative analysis methods are needed. I once again recommend the COREQ checklist to ensure you report on all the necessary criteria for qualitative studies, particularly the data analysis section. How many coders? How were themes derived? What did the coding tree entail? (etc..)

*Thank you so much for the valid comments. Details are included regarding data collectors, saturation, analysis (coding and theme), ﬁndings, and reporting (line 242-63, page 15 and line 313-20, page 20).*

Results

The first sentence of this section needs editing, as the results are not longer presented in four sections.

*Comment well taken. This section is now edited and presented on page 20 (line 322-4) and page 29 (line 404-8).*

It does seem like with so many different statistical tests, adjusting the p-value for multiple comparisons is warranted, particularly when the authors place so much emphasis on the 0.05 value as a cut-off for significance. Please adjust for these multiple comparisons or provide more detail as to why this is not necessary in your current study.

*Thank you so much for the valuable comments; comments are well acknowledged. Adjusting the p-values for multiple hypotheses tests is not always necessary. Since we report the p-values, the readers have the opportunity to make adjustments if they feel necessary. To address the issue, we added the following to the discussion section, page 36, line 561-5.*

*“Adjusting p-values is often recommended when conducting multiple hypotheses tests `simultaneously’ [1-4]. However, it is not always necessary [5-7]. As such, we did not adjust the p-value in our case as it would have increased the probability of making type II error, i.e., concluding that the intervention was not effective when it is true [8, 5, 6, 9].”*

For the qualitative quotes please cut the participant identifiers (e.g. HDA33235) or explain their significance. As they stand now they are difficult to interpret.

Please edit this section for grammar as well e.g. there is a sentence fragment “participants in Amhara (IDI11413)” after the first quote of the “enhanced the knowledge and skills of health workers” section. There are also many references to “participants” as plural after quotes- were all of these quotes said by more than one person?

*Comments are well taken and all participant identifiers are defined and grammatical errors are fixed.*

Discussion

There are still a number of typos. Please proofread carefully (e.g. “chanege" should be “change” in 5th paragraph of discussion)

*Thank you so much for the comments. The language is now edited with a native speaker and grammatically improved.*

Please define “support system” as you refer to it in this section. From the paragraph, it looks like you are referring to a strong network of implementing partners, but please be specific, as “support system” could take many different meanings.

*Thanks for the valuable comment. It is now defined (line 553-4, page 36)*

**References**

1. Tukey JW. Some thoughts on clinical trials, especially problems of multiplicity. Science. 1977;198(4318):679-84.

2. Bland JM, Altman DG. Multiple significance tests: the Bonferroni method. Bmj. 1995;310(6973):170.

3. Greenhalgh T. How to read a paper: Statistics for the non-statistician. I: Different types of data need different statistical tests. Bmj. 1997;315(7104):364-6.

4. Ludbrook J. Multiple comparison procedures updated. Clinical and Experimental Pharmacology and Physiology. 1998;25(12):1032-7.

5. Feise RJ. Do multiple outcome measures require p-value adjustment? BMC medical research methodology. 2002;2(1):8.

6. Perneger TV. What's wrong with Bonferroni adjustments. Bmj. 1998;316(7139):1236-8.

7. Rothman KJ. No adjustments are needed for multiple comparisons. Epidemiology. 1990:43-6.

8. Cole P. The evolving case-control study. The Case-Control Study Consensus and Controversy. Elsevier; 1979. p. 15-27.

9. Thomas D, Siemiatycki J, Dewar R, Robins J, Goldberg M, Armstrong B. The problem of multiple inference in studies designed to generate hypotheses. Am J Epidemiol. 1985;122(6):1080-95.
